# A bibliometric analysis of the application of stem cells in glaucoma research from 1999 to 2022

**DOI:** 10.3389/fcell.2023.1081898

**Published:** 2023-01-18

**Authors:** Yuanyuan Tao, Qian Zhang, Ming Meng, Jufang Huang

**Affiliations:** ^1^ Department of Anatomy and Neurobiology, School of Basic Medical Sciences, Central South University, Changsha, China; ^2^ Department of Neurosurgery, Xiangya Hospital, Central South University, Changsha, China

**Keywords:** stem cells, glaucoma, bibliometric analysis, hotspots, trend, VOSviewer, CiteSpace, visualization

## Abstract

**Background:** Glaucoma, a neurodegenerative disease of the retina, is the leading cause of irreversible blindness. Stem cells have therapeutic potential for glaucoma. However, few bibliometric studies have been published in this field. Concerning a visual map, this article aims to characterize the research context, cooperation relationship, hotspots, and trends concerning the application of stem cells in glaucoma research.

**Methods:** Publications focusing on stem cell research and glaucoma were retrieved from the Web of Science Core Collection. VOSviewer, CiteSpace, Microsoft Excel, and Scimago Graphica were used to map the contributions of countries or regions, authors, organizations, and journals. Journal Impact Factor data were obtained from the Web of Science Core Collection. We analyzed the tendencies, hotspots, and knowledge networks using VOSviewer, and CiteSpace.

**Results:** We analyzed 518 articles published from 1999 through 2022. In the first decade, the number of articles in this field increased slowly, and there was a marked acceleration in publication frequency after 2010. The United States, China, and England were the main contributors. Yiqin Du was the most prolific author, and among the top 10 prolific writers, Keith R. Martin’s work was cited most frequently. *Investigative Ophthalmology and Visual Science*, *Experimental Eye Research*, and *Cornea* published the most articles in this domain. The three most commonly co-cited journals were *Investigative Ophthalmology and Visual Science*, *Experimental Eye Research*, and *Proceedings of the National Academy of Sciences of the United States of America*. The Central South University, the University of Pittsburgh, and the National Institutes of Health National Eye Institute were highly prolific institutions in this research area. Our keywords analysis with VOSviewer suggested directions of future research and yielded the following recent key themes, extracellular vesicles, exosomes, mitochondria, growth factors, oxidative stress, and ocular diseases. Four co-cited references had a citation burst duration until 2022.

**Conclusion:** With improvements in overall quality of life and demographic transitions toward population aging, research and clinical focus on eye care has increased, with glaucoma as a key area of emphasis. This study added to our understanding of the global landscape and Frontier hotspots in this field.

## 1 Introduction

Glaucoma is a retinal neurodegenerative disease, that is, the second leading cause of blindness after cataracts (Quigley and Broman, 2006; Cooke and Meyer, 2015). Specific pathological features of glaucoma include optic disc atrophy and depression, retinal edge and nerve fiber layer thinning, and progressive death of retinal ganglion cells (RGCs) (Dahlmann-Noor et al., 2010; Chang and Goldberg, 2012; Karl, 2013). These changes eventually lead to progressive and irreversible visual field defects (from the periphery to the center) (Quigley and Broman, 2006). In addition to its characteristic pathological features, glaucoma’s underlying mechanisms also include axonal transport disorders at the optic disc, oxidative stress, changes in reactive glial cells, excessive activation of immune system components, activation of apoptosis and ferroptosis, loss of synaptic connections, excitotoxicity damage, mitochondrial dysfunction, and ischemia (Almasieh et al., 2012; Yao et al., 2022). The clinical manifestations of glaucoma are chronic and painless, and the associated lesions are deep within the eyeball, which poses a detection challenge during examination (McManus and Netland, 2013). Therefore, the majority of glaucoma patients are diagnosed when the disease progresses to an advanced stage with significant visual loss (Quaranta et al., 2016). Globally, over 76 million people suffer from glaucoma, approximately 8.4 million are blind, and the glaucoma prevalence is approximately 3.54% among people over 40 years (Tham and Cheng, 2015). The number of people with glaucoma is expected to reach 111.8 million by 2040 (Tham and Cheng, 2015). Glaucoma is generally classified according to the following parametersrimary or secondary, angle-open or angle-closed, and acute or chronic (Nicoara et al., 2021). Primary open-angle glaucoma is the most common type, and it is common in Europe and North America. In contrast, primary angle closure glaucoma is more common in East Asia (Kapetanakis et al., 2016).

The main risk factors for glaucoma are high intraocular pressure (IOP) and age (Quigley, 2011; Wang et al., 2020), but some glaucoma patients have IOP within normal limits (10–21 mmHg) (Almasieh et al., 2012; Li et al., 2017). Glaucoma caused by high IOP has three main treatments, drugs to reduce the generation of aqueous humor. Alternatively, surgery can be performed to introduce other outflow channels or clear obstructed areas. Thirdly, laser therapy stimulates cells to differentiate, migrate and fill damaged areas, temporarily regulating IOP (Karl, 2013; Levin, 2017). However, the above treatment is not ideal because of its limitation: for instance, it may lead to further deterioration of the disease. Treatment effects are transitory because IOP is volatile, which often necessitates repeated treatments and eye drops, which themselves are fraught with limitations, including patients’ suboptimal adherence to treatment (Tamm et al., 2013). Moreover, existing studies have found that lowering the IOP does not prevent RGC degeneration (Bull et al., 2009). Therefore, people are devoted to developing other neuroprotective strategies, especially stem cell therapy for glaucoma (Nicoara et al., 2021).

Bibliometric analysis refers to the use of quantitative techniques to analyze and visualize data pertaining to literature, which can be accomplished using software, such as VOSviewer, CiteSpace, Microsoft Excel, and Scimago Graphica (Chen, 2004; Chen et al., 2006; van Eck and Waltman, 2010; Liao et al., 2018; Moral-Muñoz et al., 2020). By means of VOSviewer and CiteSpace, we analyzed the contributions of countries or regions, authors, organizations, and journals, as well as the tendencies, hotspots, and knowledge networks. To our knowledge, there are no bibliometric studies about the application of stem cells in glaucoma research. In summary, we aimed to help researchers gain a comprehensive understanding of the past, present, and future of this field and address the following research questions (RQ) through our study.

RQ1 What is the publishing trend for articles reporting on stem cell treatment for glaucoma?

RQ2 Which are the most influential publications and primary contributing authors, institutions, journals, and countries/regions in this field?

RQ3 What is the cooperative relationship between authors, institutions, and countries/regions in this field?

RQ4 What are the research hotspots and frontier topics in this field?

## 2 Materials and methods

### 2.1 Purpose

The aims of this study were as follows: 1) analyze the basic situation and main contributors (authors, journals, institutions, and countries/regions) regarding the application of stem cells for glaucoma; 2) evaluate the cooperation relationship in this domain; 3) map the knowledge network, analyze the Frontier contents, and predict the future directions.

### 2.2 Search strategy and data collection

Web of Science (https://login.webofknowledge.com/) was used to perform the search for publications. The search and data acquisition were completed on 9 December 2022. All publications were exported in the format of “Full Record and Cited References” as plain text files. The search formula was as follows [TS= (“stem cell*” OR “progenitor stem cell*”) AND TS= (“glaucoma”)] AND DOP= [(1999-01-01/2022-12-09) AND DT= (Article OR Review)] AND LA= (English). Two investigators (YT and QZ) retrieved and screened the publications. Disagreements were discussed with a third investigator (MM) until a consensus was reached.

### 2.3 Inclusion criteria

1) Web of Science Core Collection (WoSCC)
2) Original articles and review articles
3) Written in English

### 2.4 Exclusion criteria

1) Meeting abstract, editorial materials, corrections, retractions, and book chapters
2) Unpublished papers

### 2.5 Visualized analysis

We mainly used the following software: VOSviewer (version 1.6.11, Leiden University, Leiden, Netherlands), CiteSpace (version 6.1. R1, Chaomei Chen, Drexel University, Philadelphia, PA, United States), Microsoft Excel (Redmond 2010, WA, United States). The data analysis using these applications were exported to a table that summarized the bibliometric parameters, including publication counts, publication years, total citations, average citations, co-citations, titles, countries, authors, journals, keywords, and references. The maps of co-occurrence and cooperation among authors, journals, institutions, and countries/regions, were made by VOSviewer. In the VOSviewer map, the side of the node corresponded with weighting. Links between nodes indicated cooperative relationships or common appearances in the same literature, and link thickness positively correlated with link strength. In the network visualization module, each color represented a different cluster. In the overlay visualization module, node color represented the average publication year. Blue represented the early research phase, yellow represented the recent period. We used CiteSpace to generate a keyword burst map and identify references. The red node highlighted highly cited references that co-occurred in multiple publications, and darker and lighter shades, respectively, indicated early and recent publications. In the burst module, the keywords and references were sorted by the beginning year of the burst.

## 3 Results

### 3.1 General data

**FIGURE 1:**
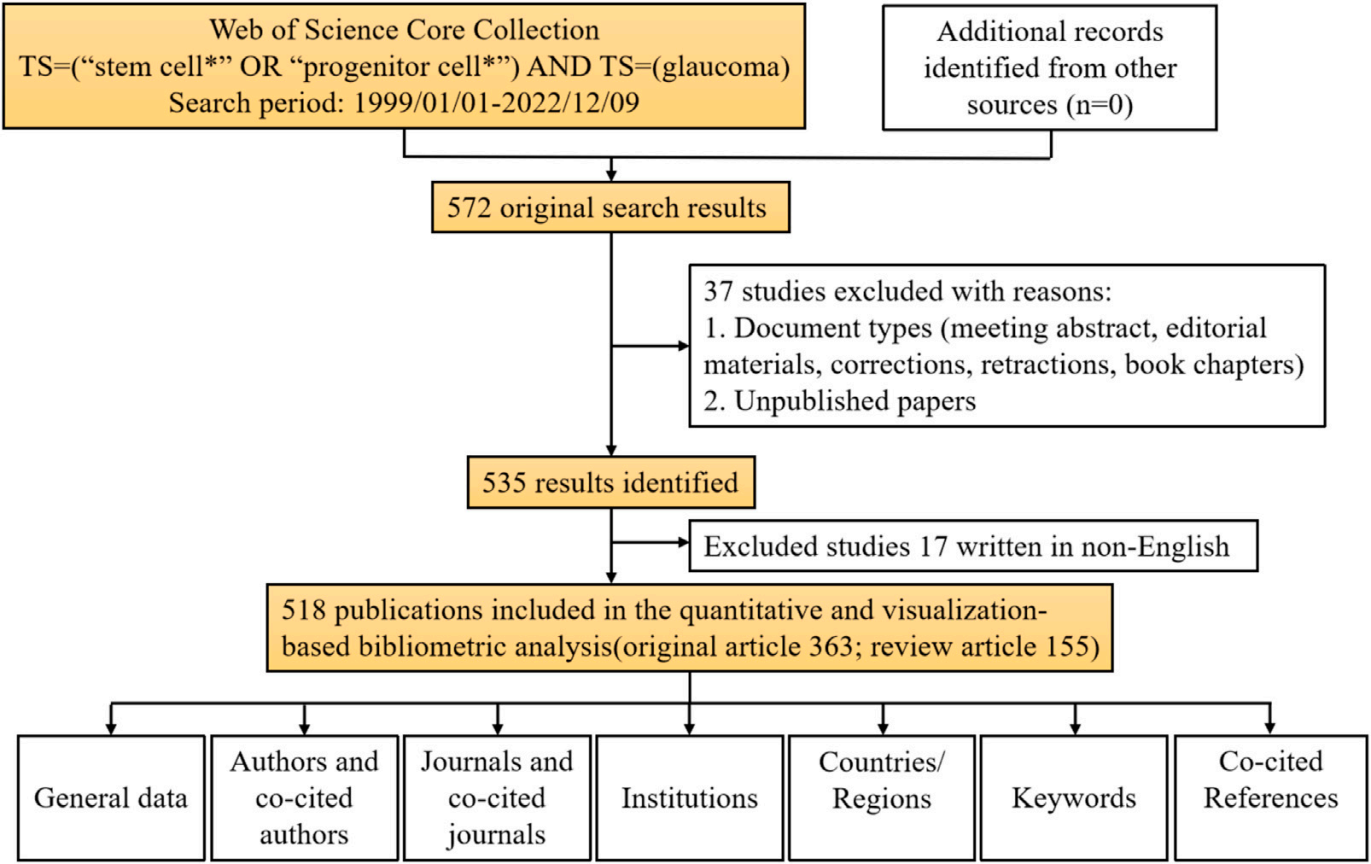
Data screening flow chart and steps of bibliometric analysis. The literature search was performed on the Web of Science Core Collection.

**FIGURE 2:**
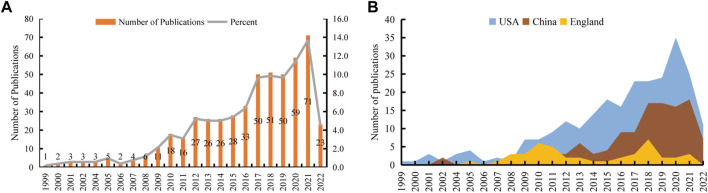
The number of publications related to the application of stem cells for glaucoma treatment and research. **(A)** The number of papers published each year. **(B)** The top three countries/regions’ annual publications.

We retrieved 572 publications from the WoSCC database, with publication dates from 1999 through 2022. Then, we limited the search results to English-language original articles and review articles. Eventually, 518 articles were brought into this analysis. There were 363 (70.08%) original articles and 155 (29.92%) review articles (Figure 1). From 1999 through 2008, the number of publications increased slowly, but there were fewer than 10 publications per year. From 2009 through 2016, the field received more attention, and the number of publications increased significantly. From 2017 through 2021, more than 50 articles in this field were published each year. Although the articles we obtained were only published before 9 December 2022, it can still be seen that the number of articles published in this field has decreased significantly in 2022. The peak publication frequency was in 2021 (*n* = 71) (Figure 2A). The general number of citations was 11069, with 21.37 citations for each paper. Overall 50 countries/regions, 2380 authors, 216 journals, and 712 institutions were represented. The United States, China, and England were the main contributors (Figure 2B).

### 3.2 Top prolific authors and co-cited authors

Most of the top 10 prolific authors in this field were from the United States (Table 1), and other authors were from China and the United Kingdom. They published 104 articles, which accounted for 20.08% of all included publications. Yiqin Du, from the University of Pittsburgh, published the most articles (*n* = 16) followed by Keith R. Martin (*n* = 14) from the University of Cambridge. We also analyzed and ranked the number of co-citations between authors. Of the top 10 co-cited authors, most were from the United States. In terms of co-citations, Harry A. Quigley, Thomas V. Johnson, and Ben Mead were ranked in the top 3. It is worth noting that Johnson ranked highly in terms of citations and co-citations among the top 10 prolific authors and top 10 co-cited authors. Supplementary Figure S1 depicts the cooperative network between authors with more than five publications included in this analysis.

**TABLE 1:** Top 10 prolific authors and co-cited authors about the application of stem cells in glaucoma.

Rank	Author	Publications	Citations	Country/Region	Co-cited author	Co-citations	Country/Region
1	Yiqin Du	16	301	United States	Harry A. Quigley	228	United States
2	Keith R. Martin	14	1010	England	Thomas V Johnson	223	United States
3	Xiaobo Xia	12	209	China	Ben Mead	170	Wales
4	Stanislav I Tomarev	11	834	United States	Deepak Ashok Lamba	104	United States
5	Natalie D. Bull	10	799	England	Yiqin Du	102	United States
6	Thomas V Johnson	9	750	United States	Robert N. Weinreb	100	United States
7	Donald J. Zack	9	255	United States	Natalie D. Bull	98	England
8	Iqbal Ahmad	8	264	United States	Gulgun Tezel	84	United States
9	Jeffrey Louis Goldberg	8	194	United States	Jason S. Meyer	80	United States
10	Ben Mead	7	521	Wales	Jorge A. Alvarado	78	United States

### 3.3 Top contributing journals

We analyzed the top 10 prolific and co-cited journals separately using VOSviewer (Table 2). A total of 216 journals published relevant articles, and 24 journals published more than five articles included in the analysis. Sorted by the number of papers, the total number of publications in the top 10 productive journals was 149 (28.76%). *Investigative Ophthalmology and Visual Science* was the most represented journal (*n* = 39; 1743 citations); *Experimental Eye Research* (*n* = 21; 483 citations) and *Cornea* (*n* = 14; 294 citations) ranked second and third, respectively. *Investigative Ophthalmology and Visual Science* (3690 co-citations) and *Experimental Eye Research* (1416 co-citations) also were the top 2 co-cited journals; *Proceedings of the National Academy of Sciences of the United States of America* (891 co-citations) ranked third in this regard.

**TABLE 2:** Top 10 prolific journals and co-cited journals on the application of stem cells in glaucoma.

Rank	Journal	Publications	Citations	If (2021)	Co-cited journal	Co-citations	If (2021)
1	Investigative Ophthalmology and Visual Science	39	1743	4.925	Investigative Ophthalmology and Visual Science	3690	4.925
2	Experimental Eye Research	21	483	3.77	Experimental Eye Research	1416	3.77
3	Cornea	14	294	3.152	Proceedings of the National Academy of Sciences of the United States of America	891	12.779
4	Scientific Reports	13	339	4.997	Ophthalmology	871	14.277
5	Progress in Retinal and Eye Research	12	721	19.704	Stem Cells	788	5.845
6	Stem Cells	11	595	5.845	Plos One	731	3.752
7	International Journal of Molecular Sciences	11	150	6.208	Journal of Neuroscience	619	6.709
8	British Journal of Ophthalmology	10	251	5.907	Progress in Retinal and Eye Research	617	19.704
9	Neural Regeneration Research	9	111	6.058	American Journal of Ophthalmology	582	5.488
10	International Journal of Ophthalmology	9	76	1.645	British Journal of Ophthalmology	555	5.907

### 3.4 Active institutions

**FIGURE 3:**
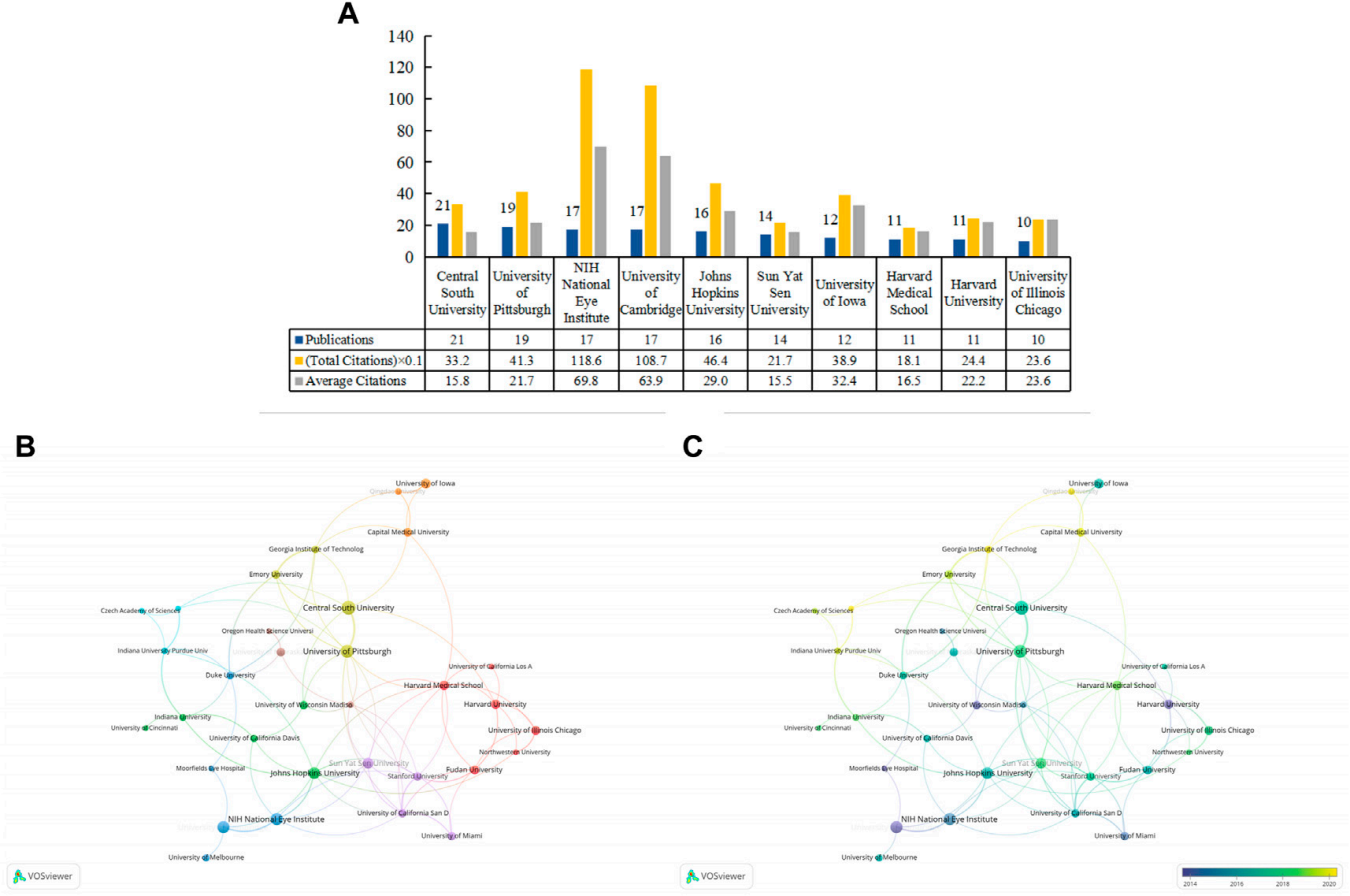
The top 10 prolific institutions and cooperative relationships between institutions in work on the applications of stem cells for glaucoma treatment and research. **(A)** The numbers of publications, total citations, and average citations in the top 10 institutions. **(B)** The cooperative network visualization map of institutions working on applications of stem cells for glaucoma treatment and research. Node size indicates the number of publications, and the thickness of the link positively correlates with the cooperation strength. **(C)** The cooperative network overlay visualization map of institutions for the application of stem cells for glaucoma treatment and research. Blue represents the early research phase, and yellow represents the recent period.

There were 712 organizations, of which 35 organizations published no less than five articles. We listed the top 10 prolific institutions (Figure 3A), eight of which were in the United States, and two were in China. Central South University ranked first (*n* = 21; 332 total citations; 15.8 average citations); University of Pittsburgh (*n* = 19; 413 total citations; 21.7 average citations) and the National Institutes of Health (NIH) National Eye Institute (*n* = 17; 1186 total citations; 69.8 average citations) followed closely behind. We generated a cooperative relationship map (Figure 3B). The University of Pittsburgh cooperated most frequently with other institutions, and it collaborated most closely with Central South University. In the overlay map, we can view the duration (in the year) each institution has published in the field (Figure 3C).

### 3.5 The contributing countries/regions

**FIGURE 4:**
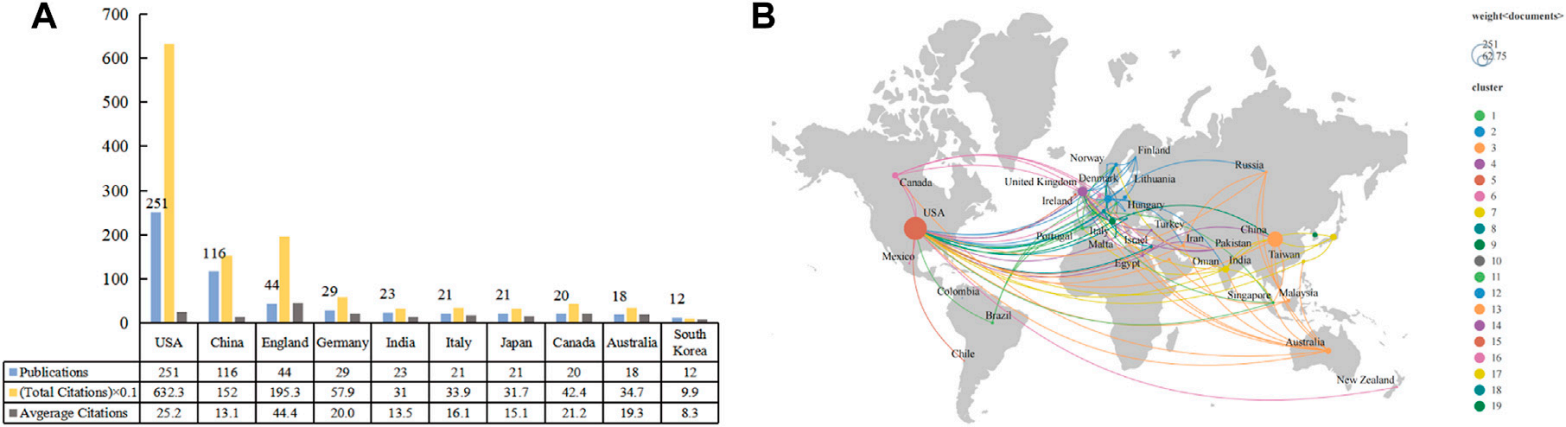
The top 10 prolific countries/regions and cooperative networks working on the application of stem cells for glaucoma treatment and research. **(A)** The number of publications, total citations, and average citations in the top 10 countries/regions. **(B)** The cooperative network visualization map of countries/regions on applications of stem cells for glaucoma treatment and research. Node size indicates the number of publications.

A total of 50 countries/regions were active in this area. There were 24 countries/regions with at least five publications included in the analysis. Figure 4A shows the top 10 countries/regions in terms of the total number of included publications. The top three countries/regions were the United States (*n* = 251; 6323 total citations; 25.2 average citations), China (*n* = 116; 1520 total citations; 13.1 average citations), and England (*n* = 44; 1953 total citations; 44.4 average citations). The total citations among the top three were markedly higher than the others. The cooperation network of these countries/regions is shown in Figure 4B. The scope of collaboration between the United States and other countries/regions was extensive, its key partners were China, England, and Germany.

### 3.6 Co-occurrence of keywords

**FIGURE 5:**
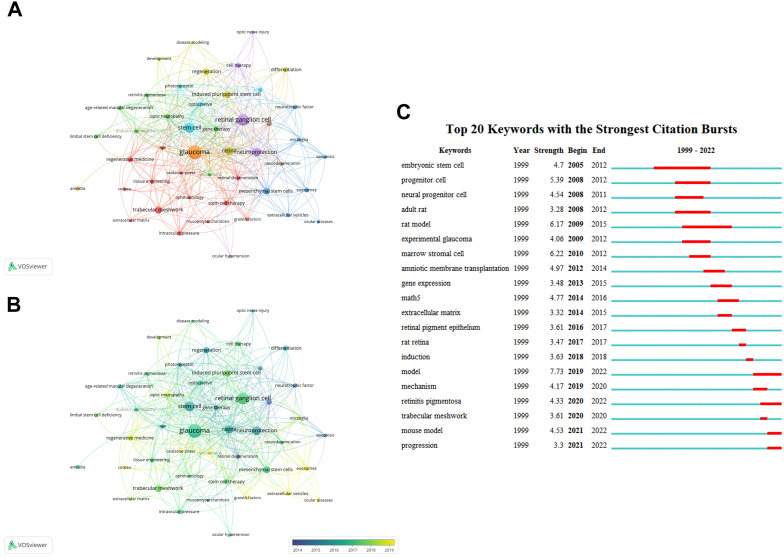
Analysis of keywords related to publications on applications of stem cells for glaucoma treatment and research. **(A)** The co-occurrence network visualization map of keywords related to the application of stem cells for glaucoma. The keywords were clustered into eight groups according to their colors. Large nodes represent keywords with high frequencies. **(B)** Keywords are colored according to their appearance for the average time. Blue represents the early stage, and yellow represents the late stage. **(C)** The top 20 keywords with the strongest citation bursts regarding applications of stem cells for glaucoma from 1999 through 2022. The red segment of the blue line denotes the burst duration of a keyword.

High-frequency keywords can reflect some knowledge domains’ evolving research frontiers (Liao et al., 2018). We identified 1157 keywords using VOSviewer, and 47 keywords appeared no less than 5 times. After excluding one word that was not associated with the keywords, we subsequently analyzed the remaining 46 keywords. There were 8 clusters (Figure 5A). “Glaucoma”, “retinal ganglion cell”, “stem cell”, “retina”, and “neuroprotection” were the top 5 keywords sorted by frequency of occurrence. In our overlay visualization map (Figure 5B), blue represents the early research phase and yellow represents the recent period. “Transplantation”, “retinal degeneration”, “neurotrophic factor”, “gene therapy”, and “apoptosis” received more attention around 2014. “Exosomes”, “extracellular vesicles”, “growth factors”, “mitochondria”, and “oxidative stress” have become the foci in recent years. CiteSpace allowed us to identify hotspots and research frontiers over time by detecting burst keywords (Chen et al., 2006). The minimum burst duration was set to 1, the red bars in Figure 5C indicate that some words have been cited frequently, while the blue bars indicate words that have been cited infrequently. The diagram exhibits the top 20 keywords with the strongest citation bursts, of which “embryonic stem cells” had the longest duration. “Model” had the highest burst strength. In general, the focus gradually deepened from “stem cell” and “experimental model” of disease to “extracellular matrix”, “retina”, and “trabecular meshwork”.

### 3.7 The cited publications and co-citation references

**FIGURE 6:**
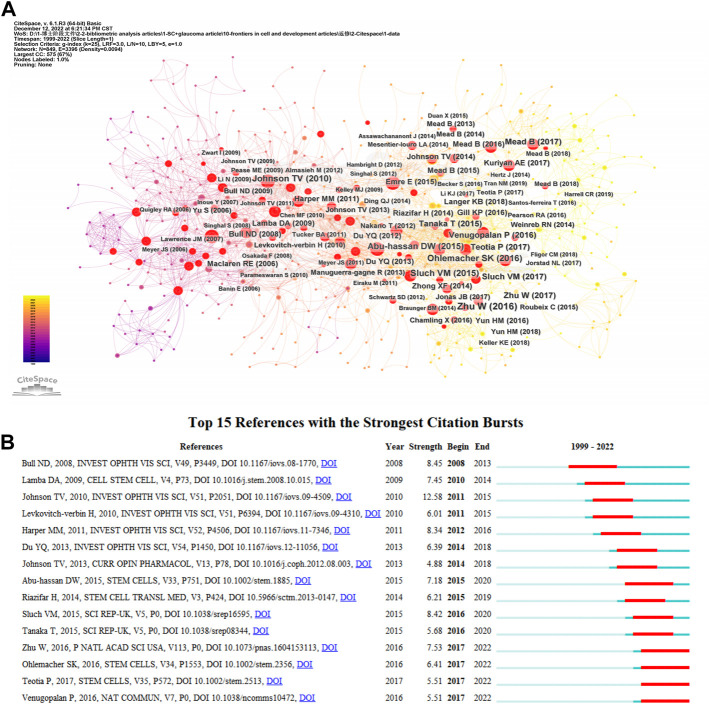
**(A)** The visual network of references to work on the applications of stem cells for glaucoma treatment and research from 1999 through 2022. **(B)** The top 15 references with the strongest citation bursts related to applications of stem cells for glaucoma between 1999 and 2022. The blue line represents the time from its first appearance to 2022, the red line represents the burst time.

Table 3 lists the top 10 cited publications. The most cited article was written by Cuenca et al. (2014), published in Progress in Retinal and Eye Research with 259 citations, titled “Cellular responses following retinal injuries and therapeutic approaches for neurodegenerative diseases”. In the article, the authors held that the injury response of retinal neurodegenerative diseases at the cellular and molecular levels is similar. Inflammatory response, oxidative stress and apoptosis may be common features of these diseases, eventually leading to cell death and retinal remodeling. In addition, the authors also summarized the molecular, anatomical and functional changes caused by damage, and the treatment were summarized. This article aimed to help researchers to establish suitable treatment methods for these pathologies (Cuenca et al., 2014). We have obtained key references with high citations in this field using CiteSpace (Figure 6A). The CiteSpace citation burst could identify references focused on by researchers during a specific period (Chen et al., 2006). There were 15 references with the strongest citation bursts when the burst duration was set to 5 years (Figure 6B). “Neuroprotective Effects of Intravitreal Mesenchymal Stem Cell Transplantation in Experimental Glaucoma” by Johnson, had the highest burst strength (*n* = 12.58) (Johnson et al., 2010). There were four articles with citation bursts ending in 2022 that indicated they had received more attention in recent years (Ohlemacher et al., 2016; Venugopalan et al., 2016; Zhu et al., 2016; Teotia et al., 2017).

**TABLE 3:** Top 10 most cited papers related to the application of stem cells in glaucoma.

Rank	Title	Journal	Type	Corresponding author	Affiliation	Year	Citations
1	Cellular responses following retinal injuries and therapeutic approaches for neurodegenerative diseases	Progress in Retinal and Eye Research	Article	Nicolas Cuenca	Universitat d'Alacant	2014	259
2	Neuroprotective Effects of Intravitreal Mesenchymal Stem Cell Transplantation in Experimental Glaucoma	Investigative Ophthalmology and Visual Science	Article	Keith R. Martin	University of Cambridge	2010	224
3	Bone Marrow-Derived Mesenchymal Stem Cells-Derived Exosomes Promote Survival of Retinal Ganglion Cells Through miRNA-Dependent Mechanisms	Stem Cells Translational Medicine	Article	Ben Mead	NIH National Eye Institute	2017	201
4	Identification of retinal ganglion cell neuroprotection conferred by platelet-derived growth factor through analysis of the mesenchymal stem cell secretome	Brain	Article	Thomas V Johnson	Johns Hopkins Medicine	2014	116
5	Neurotrophin-Induced Differentiation of Human Embryonic Stem Cells on Three-Dimensional Polymeric Scaffolds	Tissue Engineering	Article	Robert Langer	Massachusetts Institute of Technology	2005	110
6	Development and Characterization of an Adult Retinal Explant Organotypic Tissue Culture System as an *In Vitro* Intraocular Stem Cell Transplantation Model	Investigative Ophthalmology and Visual Science	Article	Keith R. Martin	University of Cambridge	2008	107
7	Transplantation of BDNF-Secreting Mesenchymal Stem Cells Provides Neuroprotection in Chronically Hypertensive Rat Eyes	Investigative Ophthalmology and Visual Science	Article	Matthew M. Harper	US Department of Veterans Affairs Veterans Health Administration (VHA) Iowa City VA Healthcare System	2011	100
8	Differentiation of human ESCs to retinal ganglion cells using a CRISPR engineered reporter cell line	Scientific Reports	Article	Donald J. Zack	Johns Hopkins University	2015	99
9	Mesenchymal stem cell-derived extracellular vesicles and retinal ischemia-reperfusion	Biomaterials	Article	Sriram Ravindran	University of Illinois	2019	98
10	Transplantation of Mesenchymal Stem Cells Promotes Tissue Regeneration in a Glaucoma Model Through Laser-Induced Paracrine Factor Secretion and Progenitor Cell Recruitment	Stem Cells	Article	Denis-Claude Roy	Universite de Montreal	2013	94

## 4 Discussion

The growth or decline in the number of academic publications in a particular field is a good indicator of overall trends (Peng et al., 2020). Through the analysis of the publications in this field, we found that the research was increasing year by year, suggesting that this field will still be a focus for researchers in the future. In the past decade, the number of published papers has increased significantly; this could be due to the globally aging population and growing awareness of eye healthcare (Cheng et al., 2020a). We analyzed 518 articles published from 1999 through 2022, that met the inclusion criteria, and these publications cover 50 countries/regions, 712 institutions, and 2380 authors, which means that the application of stem cells for glaucoma treatment has attracted the attention of researchers worldwide. The contribution of the United States in this field is particularly prominent, which may be related to the fact that patients with primary open-angle glaucoma are mostly located in North America (Zhang et al., 2021). Another important contributor is China, which may be related to the aging demographic pyramid in China (Wan et al., 2020). Among the top 10 prolific institutions, eight institutions are in developed countries and two institutions are in developing countries, indicating that developed countries pay more attention to eye health. The University of Pittsburgh, in the United States, cooperated most frequently with other organizations, and it cooperated most closely with Central South University in China. Yiqin Du, based in the United States, had the most publications. These results indicate that the UAS occupies a dominant position in this field. In terms of the total number of publications and citations. *Investigative Ophthalmology and Visual Science* (*n* = 39; 1743 citations) and *Experimental Eye Research* (*n* = 21; 483 citations) were the top two journals in this field. *Progress in Retinal and Eye Research* is the most influential of the top 10 prolific journals, with 60.08 citations per article, it is also ranked as Q1 by the Journal Citation Reports. Our analysis identifies the classical and important contents in this field, and researchers can quickly establish a knowledge architecture guided by these data.

We can determine the information that investigators emphasize from an analysis of references. CiteSpace was used to analyze references, and 25148 references were discovered. The minimum burst duration in the burstness module was set to five, and we listed the top 15 references (Figure 6B). Additionally, we analyzed the references at the red nodes (Figure 6A). By means of cluster analysis, 10 clusters are shown (Supplementary Figure S2), including “gene therapy”, “retinal ganglion cell”, “marrow-derived mesenchymal stem cell”, “optic neuropathies”, “cell-derived small extracellular vesicle”, “neurotrophic factor delivery”, “patient-specific stem cell”, “trabecular meshwork cell”, “survival differentiation”, and “ocular disease therapeutics”. Development speed is still relatively slow throughout the field. The research background of this domain can be divided into four stages, according to the theme by analyzing the literature with red nodes.

### 4.1 Phase I: Cell differentiation and replacement

Researchers focused on the simple differentiation and replacement of cells before 2009. The types of cells emphasized during this period are also relatively complex, including various stem cells and cells with similar activity (such as Müller cells and TM cells) (Lawrence et al., 2007; Bull et al., 2008; Kelley et al., 2009). Some studies have shown that when human embryonic stem cell-derived neural progenitor cells (NPCs) or retinal cells are transplanted into the subretinal space, they survive for some time, and a few cells could integrate into the retina where they could further differentiate into retinal cells (Banin et al., 2006; Meyer et al., 2006; Lamba et al., 2009). Conditions for inducing embryonic stem cells from mice, monkeys, and humans to differentiate into rod and cone cells have been investigated (Osakada et al., 2008). Another type of cell is bone marrow mesenchymal stem cells, scholars have confirmed that by transplanting this cell, it could be differentiated and partially integrate into the RGC layer to reduce retinal damage (Yu et al., 2006; Inoue et al., 2007; Li et al., 2009). Moreover, other studies have used Müller cells and oligodendrocyte precursor cells (Lawrence et al., 2007; Bull et al., 2008; Bull et al., 2009; Kelley et al., 2009).

### 4.2 Phase II: Stem cell-derived neurotrophic factors

From 2009 to 2013, there was growing concern that the effects of stem cell transplantation on the retina may be related to its secretion of neurotrophic factors. Pease et al. (2009) injected an adeno-associated viral vector that contained ciliary-derived neurotrophic factor (CNTF) and brain-derived neurotrophic factor (BNTF) directly into the vitreous body of glaucomatous rats. They found that CNTF had an obvious neuroprotective effect, emphasizing that the action of neurotrophic factors on damaged RGCs are determined by the delivery method, dose, and model of disease (Pease et al., 2009). Several studies have confirmed that bone marrow or dental pulp mesenchymal stem cells, in addition to integration, and differentiation, provide neurotrophic factors to the damaged retina (Johnson et al., 2010; Levkovitch-Verbin et al., 2010; Harper et al., 2011; Johnson and Martin, 2013; Manuguerra-Gagne et al., 2013; Mead et al., 2013; Johnson et al., 2014). Cell therapy can supply more durable, low-dose, multi-factor better protection compared with direct supplementation of nutritional factors (Eiraku et al., 2011; Nakano et al., 2012). Also, the release of neurotrophic factors in cell therapy is based on the host’s retinal condition with less trauma, and it can be engineered to allow donor cells to express specific factors. Concerns have also been raised over the details of how to differentiate stem cells into RGCs (Chen et al., 2010; Meyer et al., 2011; Tucker et al., 2011). During this period, attention began to be paid to the role of induced pluripotent stem cells (iPSCs) and differentiated stem cells in the trabecular meshwork in improving IOP (Kelley et al., 2009; Du et al., 2012; Du et al., 2013).

### 4.3 Phase III: Induction of human pluripotent stem cells

Between 2014 and 2016, the induction of human pluripotent stem cells became a research hotspot. This was seen as a way to avoid both the ethical issues caused by embryonic stem cells and the host immune rejection. Researchers induced pluripotency in cells and used them to refill damaged areas (Ding et al., 2014; Zhong et al., 2014; Abu-Hassan et al., 2015; Zhu et al., 2016). They also explored the molecular details of iPSCs differentiation and identified differentiated cells (Riazifar et al., 2014; Ohlemacher et al., 2016), and then people gradually focused on targeted induction of IPSC to differentiate into cells with specific functions, such as using CRISPR technology (Sluch et al., 2015; Tanaka et al., 2015; Teotia et al., 2017). Additionally, there are still some researchers continuing to explore the different sources of mesenchymal stem cells (MSCs) and their secretion of nutritional factors on the role of retinal (Mead et al., 2014; Mesentier-Louro et al., 2014; Roubeix et al., 2015).

### 4.4 Phase IV: Cell-free therapy

There was a growing interest in a cell-free treatment approach from 2017 to 2022 (Phan et al., 2018; Rahmani et al., 2020; Han et al., 2021). Exosomes are vesicle structures containing a variety of ribonucleic acids (RNAs) and proteins with diameters of 40–100 nm (Hobor et al., 2018; Cheng et al., 2020b). Mead and Tomarev (2017) have made an outstanding contribution to stem cell therapy for glaucoma. They transported exosomes extracted from bone marrow mesenchymal stem cells (BMSCs) into the eyes, which were found to accelerate the regeneration of RGCs and their axons, and this therapeutic effect was weakened after knocking out Argonaute2, which is a kind of microRNA (miR) for the first time (Mead and Tomarev, 2017). They demonstrated for the first time that BMSC-derived exosomes may exert a protective action on RGCs through miRNAs (Mead and Tomarev, 2017). Exosomes effectively avoid a variety of complications in cell therapy, such as immune rejection, tumorigenesis, and difficulties in integrating with host tissue. Exosomes have become a potential resource for cell and gene therapy due to their small size, easy storage, and non-proliferation (Lu et al., 2019; Feng et al., 2021). Mesenchymal cells can produce more exosomes than other cells (Yeo et al., 2013; Phan et al., 2018).

Because of the remarkable advantages of cell-free therapy, may be the reason for the significant decrease in the research on stem cell therapy for glaucoma in 2022, as the shifts in a more optimized direction. Increasing attention to the use of extracellular vesicles as therapeutic agents or drug carriers (Anand et al., 2022; Sanie-Jahromi et al., 2022). The role of exosome content gradually emerges. Yu et al. (2022) found that tumor necrosis factor-α mediates the neuroprotective effect of miR-21-5p-rich exosomes in glaucoma through the maternally expressed gene 3/programmed cell death four axis. Studies have shown that miR-143-3p, miR-125b-5p, and miR-1260b in aqueous humor may be therapeutic intervention targets, and microRNA can be an early biomarker or therapeutic strategy by regulating its level (Martinez and Peplow, 2022). RGCs are a group of neurons located at the distal end of the eye, their axons contain large amounts of mitochondria (Anand et al., 2022). There are studies found that mitochondrial dysfunction or abnormal mitophagy is involved in glaucomatous damage and affects neurogenesis and neuronal viability (Rosignol et al., 2020; Ozgen et al., 2022). At the same time, some researchers were paying increasing attention to the genetic aspects of the field. Wei et al. (2022) discovered key genes that regulate self-renewal and differentiation of NPCs by using 5-(p-hydroxyphenyl)-1,2-dithione-3-thione to treat mammalian NPCs to promote their differentiation into neurons and oligodendrocytes and found that mRNA and protein expression of key genes regulating NPCs self-renewal and differentiation decreased, such as β-catenin. Optineurin (E50K) mutant astrocytes caused neurodegeneration in healthy RGCs (Gomes et al., 2022). Xue et al. (2022) investigated the common genetic causes of age-related macular degeneration, diabetic retinopathy, glaucoma, retinal detachment, and myopia, they found that genes associated with common genetic sites were involved in neuronal differentiation and eye development systems. Single-cell RNA sequencing data showed that their gene expression from pluripotent progenitor cells into retinal cells increased during retinal development (Xue et al., 2022).

Notably, iPSCs still received attention in 2022 (Coulon et al., 2022). The trabecular meshwork cells can mediate the regulation of intraocular pressure by regulating aqueous humor flow (Zhu et al., 2017). Wang et al. (2022) designed a nanoparticle to label trabecular meshwork cells, and the use of nanoparticles not only facilitated long-term tracking, but also improved the transmission accuracy of transplanted cells *in vivo* experiments (Wang et al., 2022). It is more important that the use of magnet temporarily enhanced the effectiveness of cell-based therapy in glaucoma-related pathology, and promoted the clinical transformation of stem cell-based glaucoma therapy (Zhu et al., 2016; Wang et al., 2022).

### 4.5 Research frontiers

A total of 47 keywords with no less than five occurrences were identified and divided into 8 clusters. The top 3 clusters are red (13 items), green (7 items), and blue (7 items) in Figure 5A. It is worth noting that some studies have focused on the neuroprotective role of mesenchymal stem cells in damaged RGCs through extracellular vesicles (exosomes, microscopic vesicles) (Mead et al., 2018; Mathew et al., 2019; Mead et al., 2020; Mathew et al., 2021), which may also involve apoptosis and glial cell-mediated inflammatory responses (Alghamdi et al., 2020; Yu et al., 2021). According to the analysis in the overlay visualization module, research on exosomes, extracellular vesicles, and oxidative stress in this field have been a hot topic in the last few years (Mathew et al., 2021; Reboussin et al., 2022; Yu et al., 2022). The following three themes may be worthy of further study in the future: 1) the neuroprotective effect of mesenchymal stem cell-derived exosomes in RGCs, 2) growth factors, MSCs, and iPSCs in regenerative medicine for glaucoma, and 3) oxidative stress involving in the injury mechanism of glaucoma.

### 4.6 Limitations

First, because the visualization software has limitations on the format of the data, we only collected text data from the WoSCC. However, as the most widely used database in the world, the WoSCC met our analytical demands (Shi et al., 2022). Second, we adopted English publications and limited their types, and we only analyzed original articles and reviews. Third, the indicators related to citations will change over time. We should integrate various indexes to analyze Journal Impact Factor, Source Normalized Impact per Paper, and SCImago Journal Rank (Deng et al., 2020). Fourth, updating the database may affect our results, but this effect should be slight.

## 5 Conclusion

With the improvements in quality of life and population aging transitions, followed by an increase in the number of neurodegenerative diseases, such as glaucoma (Aishwarya et al., 2022). There is an urgent need for an effective, fewer side effects treatment of glaucoma. Stem cells provide the possibility for its treatment. This field has been explored for nearly 20 years. Our research enables readers to quickly obtain the guiding information in this field through visual maps while maintaining the objectivity and accuracy of the information. This study summarized and visualized the global trends, cooperative relationships, hotspots, and Frontier topics about the application of stem cells in glaucoma treatment. In the future, researchers explore the mechanisms and clinical trials.

## Data Availability

The original contributions presented in the study are included in the article/Supplementary Material, further inquiries can be directed to the corresponding author.

## Supplementary material

Supplementary Figure S1 The cooperative relationship between authors. **(A)** The co-authorship network visualization map of authors related to work on applications of stem cells for glaucoma. The authors were clustered into 3 groups according to their color. Large nodes represent prolific authors. **(B)** In the overlay visualization map between authors, the weight is the number of publications, and the color is the average publication year. Blue represents the early stage, and yellow represents the late stage.

Supplementary Figure S2 The cluster analysis of the highly cited references.
