# Supplementary figures and images for: A bibliometric analysis of the application of stem cells in glaucoma research from 1999 to 2022
Source: Front Cell Dev Biol. 2023 Jan 18;11:1081898. doi: 10.3389/fcell.2023.1081898 (PMC9889543; doi:10.3389/fcell.2023.1081898)

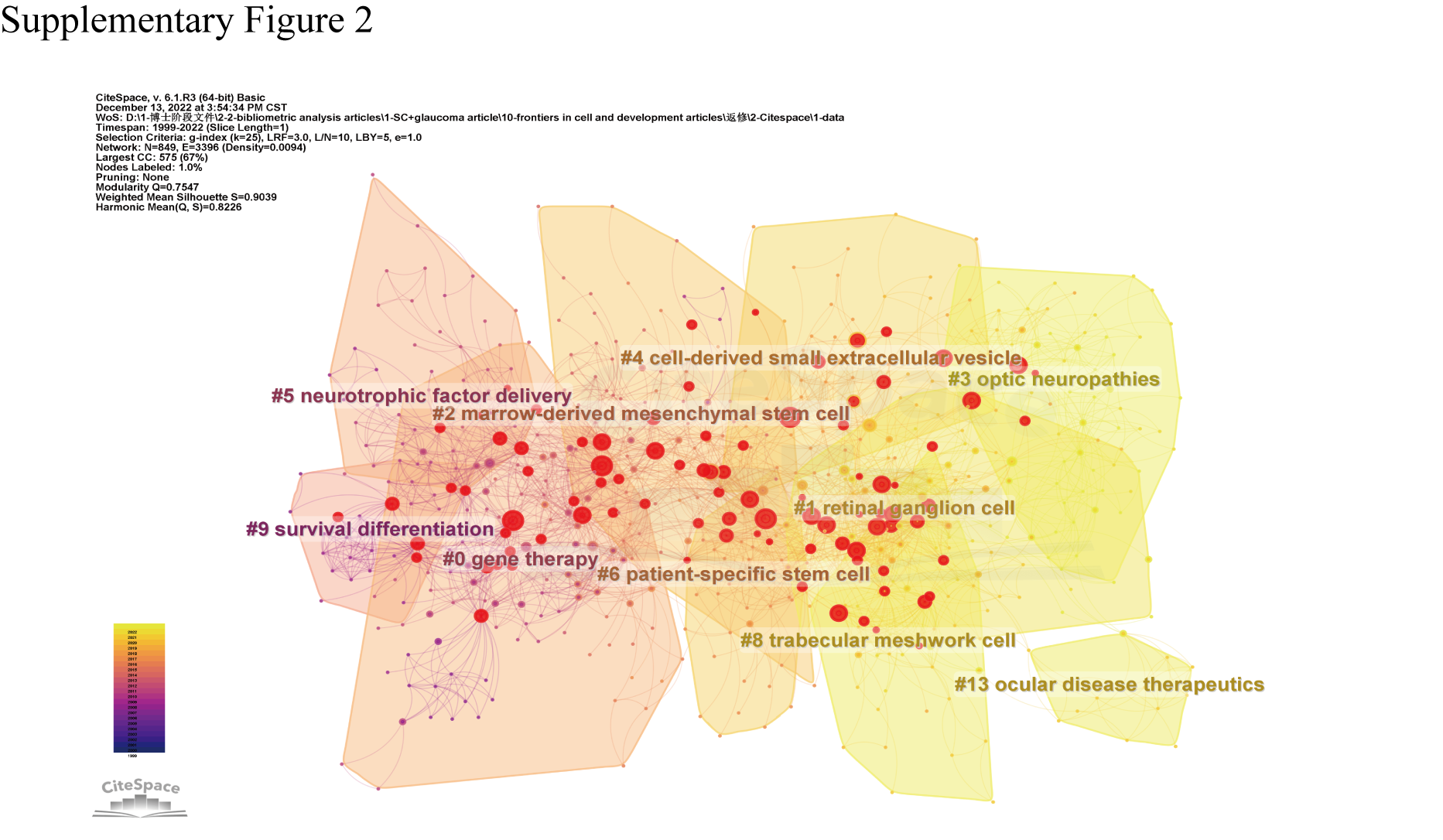

Supplement: Supplementary file 1 [file Image2.TIF]

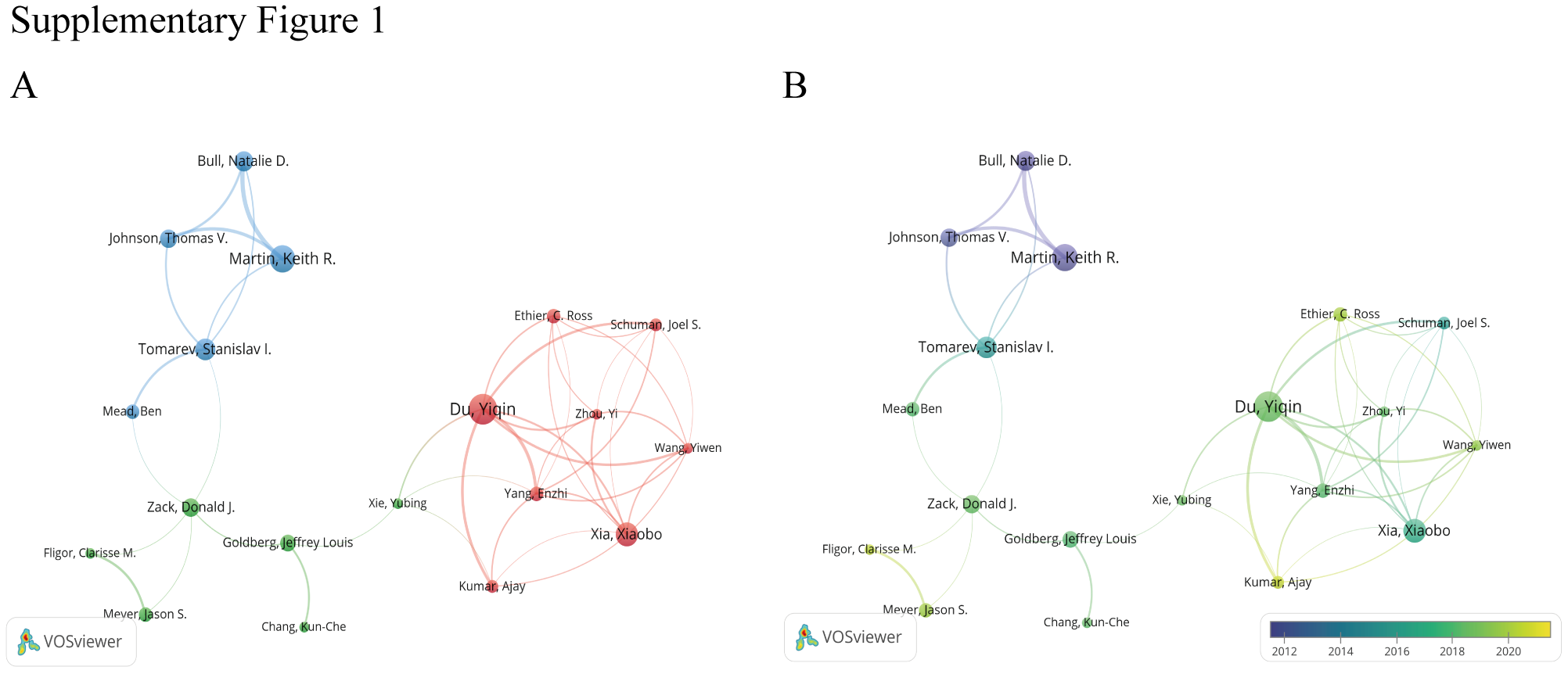

Supplement: Supplementary file 2 [file Image1.TIF]
